# Glycine and GABAA receptors suppressively regulate the inspiratory-related calcium rise in the thoracic inspiratory cells of the neonatal rat

**DOI:** 10.1186/s12576-022-00850-4

**Published:** 2022-10-03

**Authors:** Yoshihiro Mikami, Makito Iizuka, Hiroshi Onimaru, Masahiko Izumizaki

**Affiliations:** 1 Department of Physiology, Showa University School of Medicine, 1-5-8 Hatanodai, Shinagawa-ku, Tokyo, 142-8555 Japan; 2 Department of Orthopaedic Surgery, Showa University Hospital, 1-5-8 Hatanodai, Shinagawa-ku, Tokyo, 142-8555 Japan

**Keywords:** Inspiratory motor activity, Spinal cord, Inhibitory interneuron, Neonatal rat

## Abstract

We previously demonstrated that in an isolated brainstem–spinal cord preparation from neonatal rats, a local bath application of strychnine (a broad antagonist of glycine and GABA_A_ receptors) to the spinal cord enhances thoracic inspiratory motor activity. Herein, to investigate the involvement of the inspiratory spinal interneurons that provide excitatory input to the motoneuron, we conducted calcium imaging using this preparation. Oregon Green 488 BAPTA-1 AM, a fluorescent calcium indicator, was injected into the ventromedial surface of the thoracic cord. In all cells that showed inspiratory-related fluorescence changes > 2% of the baseline fluorescence intensity, the inspiratory-related fluorescence change decreased when the focal depth was deepened. The application of strychnine to the spinal cord increased the inspiratory-related intracellular calcium rise in these cells. These results suggest that the enhancement of inspiratory interneuron activity could be involved in this enhancement of inspiratory motor activity.

## Background

Breathing movements are achieved by the activity of numerous motoneurons that are distributed along the cervical and thoracic spinal cord and fire in proper spatial and temporal sequences [1]. Respiratory motoneurons in the thoracic spinal cord innervate the ribcage muscles. The contraction of the external intercostal muscles and other inspiratory ribcage muscles displaces the ribs and increases the volume of the ribcage [1]. Although the spinal interneurons are believed to play a role in the modulation of the thoracic inspiratory motor activity, the detailed mechanisms remain unknown. Our previous study demonstrated that the inspiratory motor activity in the neonatal rat rostral thoracic ventral root was enhanced when glycine and γ-aminobutyric acid type A (GABA_A_) receptors in the thoracic spinal cord were blocked by a local bath application of the receptors' antagonist, strychnine [2]. Using a voltage-sensitive dye, we further observed that this blockade of glycine and GABA_A_ receptors increased inspiratory depolarizing signals in the interneuron area of the rostral thoracic spinal cord [2]. Using whole-cell patch clamp recording and the in situ hybridization method, we also showed that some inspiratory interneurons in the ventromedial portion of the rostral thoracic spinal cord are glutamatergic [3]. We have thus hypothesized that the enhancement of inspiratory motor activity comes from the enhancement of firing in these active inspiratory interneurons and/or the recruitment of inspiratory interneurons that give excitatory synaptic input to the motoneurons.

Confocal fluorescence microscopy was developed to minimize the out-of-focus haze of fluorescent objects [4]. In combination with calcium fluorescence indicators, confocal fluorescence microscopes have been used to monitor intracellular calcium changes in individual neurons and astrocytes in various regions of the brain [5]. Several studies in the field of respiratory rhythm and pattern generation have used this method to examine brainstem cells [6–9]. One of the studies revealed that many cells in both the ventral respiratory column (VRC) and the pre-Bötzinger complex (preBötC) exhibited a rise in intracellular calcium in phase with the inspiratory activity [9], and another study reported that not only neurons but also many putative astrocytes in the preBötC exhibited a respiratory-related calcium rise [6]. Over 60% of Phox2B-positive neurons in the parafacial respiratory group (pFRG) showed a respiratory-related calcium rise [7]. However, in all of these studies, the effects of out-of-focus fluorescence were not examined. Since respiratory networks are distributed in three dimensions, the effects of cells located in different focus planes should be considered.

The main objectives of our present investigation were thus to (i) monitor the inspiratory-related calcium rise in the ventromedial portion of the thoracic spinal cord at the single-cell level using confocal microscopy and (ii) examine the possibility that the disinhibition-induced increase in inspiratory thoracic motor activity is caused by increased activity of active inspiratory neurons and/or a recruitment of inactive inspiratory neurons. We also examined the effects of the focal depth on respiratory-related calcium signals to ensure that fluorescence signals come from the cells of interest. The preliminary results were presented in abstract form [10].

## Materials and methods

### Ethical approval

This study was approved by the Animal Research Committee of Showa University, which operates in accord with the Japanese Government’s Law No. 105 for the care and use of laboratory animals.

### Preparation

**Fig. 1:**
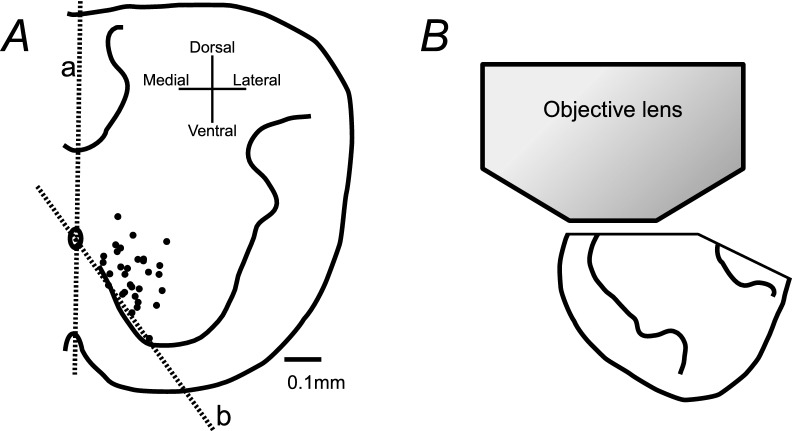
Schematic diagram explaining the method used to observe cells in the neonatal rat thoracic spinal cord. **A** Distribution of inspiratory interneurons in the cross-sectional plane of the thoracic spinal cord modified from Fig. 5 in Iizuka et al. [3]. Each interneuron is indicated by a *black circle*. The spinal cord from T1 to T7 was sectioned at the midline indicated by *dotted line a*. As shown by *dotted line b*, the ventral funiculus was carefully removed with insect pins. **B** Arrangement of the preparation for the objective lens

We used 0–2-day-old Wistar rats. Under deep anesthesia with isoflurane, the rats were decapitated and moved to a dissection chamber filled with modified Krebs solution (composition below). The brainstem and spinal cord were isolated as described [2, 3]. The thoracic cord was hemisected from the first to the seventh thoracic segments, and the left or right side was removed. To expose cells in the medioventral region, the ventral funiculus was carefully removed using a fine stainless steel insect pin (Fig. 1A). The chamber was split at the level of the first to third cervical segments to separate the preparation into the brainstem and spinal cord parts in three preparations [2].

Each compartment was continuously superfused at 2 to 3 ml/min with a modified Krebs solution containing (mM): 124 NaCl, 5.0 KCl, 1.2 NaHPO_4_, 2.4 CaCl_2_, 1.3 MgCl_2_, 26 NaHCO_3_, and 30 glucose (pH adjusted to 7.4 by gassing with 95% O_2_ and 5% CO_2_) at 25–27 °C. In the experiment split into two compartments, strychnine (10 μm) was applied to the caudal compartment to block the glycine and GABA_A_ receptors within the spinal cord.

### Optical recording of intracellular calcium changes

The intracellular calcium changes were optically recorded using a fluorescent cell-permeant calcium indicator, Oregon Green (Oregon Green 488 BAPTA-1 AM, Molecular Probes, Eugene, OR, USA). First, 200 μm Oregon Green was solved with 10% Pluronic^®^ F-127 (Molecular Probes) in dimethyl sulfoxide (DMSO; Wako, Osaka, Japan) and was injected into the ventromedial surface of the thoracic cord using a fine glass pipette (o.d. of glass: 1.0 mm, i.d.: 0.75 mm, cat. no. TW100F-4, World Precision Instruments, Sarasota, FL). The tip of the glass pipette was broken by pushing it against a pin placed near the preparation in the experimental chamber (pipette tip o.d.: 5–20 μm), and was inserted 0.05–0.15 mm deep from the surface, then the Oregon Green was injected using pressure connecting a polyethylene tube (o.d.: 1.2 mm, i.d.: 0.8 mm, approx. 40 cm long) and a 10 ml syringe.

Changes in the fluorescence of the calcium indicator were detected by a Nipkow-disc confocal microscope system (confocal scanner unit: CSU-W1, Yokogawa, Japan; upright microscope: BX51WI, Olympus, Tokyo; objective lens: LUMPLFLN40XW, Olympus; electron multiplying CCD camera: iXon Ultra, Andor Technology, Belfast, Northern Ireland, UK). The CCD-based camera head has a 13.312 × 13.312 mm imaging area consisting of 1024 × 1024 pixels. The final magnification was adjusted by × 40 times, so that an area of 0.3328 × 0.3328 mm was covered by the image sensor. The fluorescence intensity was obtained using the live cell imaging software program iQ3 (Andor Technology). The fluorescence value of each pixel ranged from 0 to 65,536. The focus of the microscope was set on the ventromedial surface of the thoracic cord (Fig. 1B). The fluorescence was recorded with an exposure time of 150–200 ms and an acquisition time of 200–300 ms/frame for 480–720 frames.

### Electrophysiological recordings

For the analysis of the relationship between the changes in intracellular calcium and respiratory motor activity, the inspiratory motoneuron activity was monitored at the fourth cervical ventral root (C4VR) with a glass capillary suction electrode. The obtained activities were amplified (× 10,000) and bandpass filtered (0.5–1000 Hz). Each electrical signal was digitized at 4 kHz (Powerlab, AD Instruments, Sydney, Australia) and stored on a personal computer using Chart v7.0/s software (AD Instruments). The shutter timing of the CCD-based camera was also recorded.

### Data analysis

**Fig. 2:**
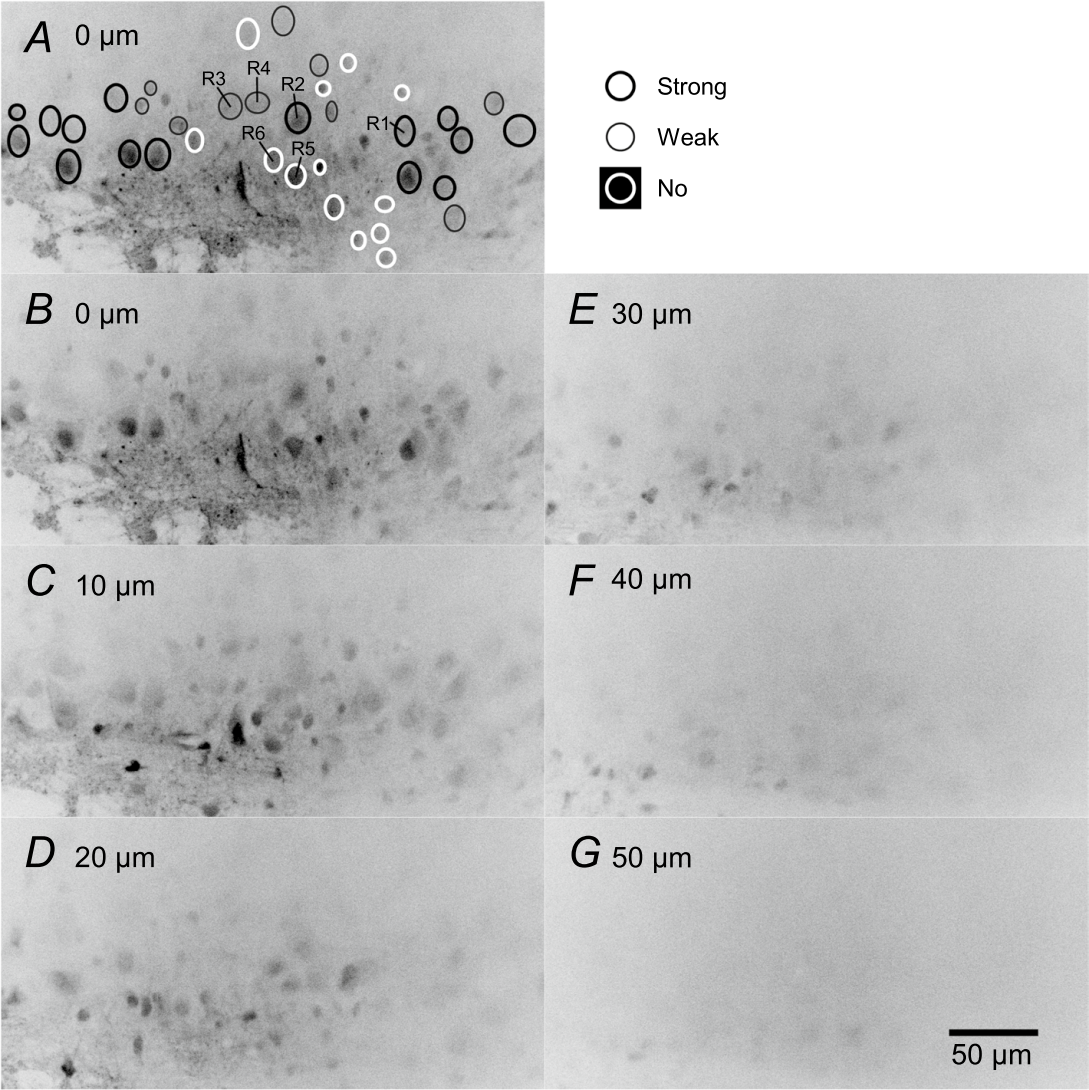
Effects of focal depth on the fluorescent image. **A**, **B** Same fluorescence image was obtained when the focal plane was set near the surface. This focal depth was set at 0 μm. Regions with higher fluorescence intensity are shown in *darker colors* in these fluorescence images. At this focal plane, many elliptical regions darker than the peripheral regions were visible. Each dark elliptical region was regarded as a single cell, and the outline of each cell was selected using the ellipse tool (A). A total of 38 regions were selected and classified into three groups based on their amplitudes of inspiratory-related fluorescence changes: strong (*n* = 15), *thick black line*; weak (*n* = 10), *thin black line*; and no (*n* = 13), *thick white line* inspiratory-modulated groups (see the Results for the group definitions). Raw and averaged fluorescence signals in regions 1 to 6 (labeled *R*1 to *R*6 in panel A) are shown in Figs. 4 and 5. **C**–**G** Fluorescence images obtained from the same area in panel B, when the focal depth was 10, 20, 30, 40, and 50 μm deeper than the focal plane in panel B, respectively

Greater fluorescent intensity is presented as a darker color in the fluorescence images. As in earlier studies [6, 11], the cells that were stained with Oregon Green had oval shapes. An ellipsoid region that was darker than the surrounding region was considered a single cell, and we used an ellipse tool to select the outline of each cell (see Fig. 2A). The mean fluorescence intensity in each region was calculated and saved in an XLD format file. The corresponding electrophysiological data were saved as a text file.

For the analysis of the relationship between fluorescence changes and respiratory motor activities, we created a program that merges an XLD file and a text file into a single WAV format file with the C program (CreaTact, Ibaraki, Japan). To obtain the same sampling rate as the electrophysiological data (i.e., 4 kHz), single fluorescent data points and their next fluorescent data point were connected by a line and interpolated at 4 kHz. Each data point of each channel was divided by the maximum value of each channel and was multiplied by 64,000. This procedure was performed to transfer the data to an analysis program, Spike2 (CED, Cambridge, England), and the maximum value of each channel became 4.8826599 as a result. As mentioned above, the fluorescence value of each pixel ranged from 0 to 65,536. Since the baseline fluorescence was highly dependent on the degree of staining, there was a significant difference in fluorescence values among regions. Since this procedure always produced 4.8826599 as the largest fluorescence value and 0 as the zero-fluorescence value, the vertical axis range could collectively be adjusted on the Spike2 window.

To detect inspiratory-related fluorescence changes, we averaged the fluorescence signal with reference to the inspiratory burst activity in C4VR. The electrical activity in C4VR was DC-removed, fully rectified, and integrated with the time constant of 0.1 s. The onset or peak of this leaky integrated C4 wave was then used as a trigger. In all experiments, more than five C4 inspiratory bursts were used to obtain the mean value. We assumed that the period from 5.0 to 1.5 s before the trigger would be the expiratory phase and that there was no inspiratory activity; therefore, the mean fluorescence value during this period in each region was defined as the baseline value for the region.

Since the peak of the inspiratory fluorescence signal should occur within ± 0.5 s of the trigger, we selected the maximum fluorescence value in this 1-s period. The amplitude of the inspiratory-related fluorescence signal was defined as the value obtained after the subtraction of the baseline value from this maximum fluorescence value. When the amplitude of the inspiratory-related fluorescence signal was greater than the sixfold standard deviation (6SD) of the baseline value, we judged that the region showed significant inspiratory-related fluorescence changes. There were 4000 data points during this 1-s period of the averaged fluorescence data. When these data points are assumed to show a normal distribution, the probability that the amplitude is greater than 6SD will be 7.892 × 10^−6^ (1.973 × 10^−9^ multiplied by 4000). The amplitudes of the fluorescence change are expressed as the percentage ratio to the baseline.

Values are presented as mean ± SD. Statistical differences between the two groups were examined using the paired *t* test. Statistical differences between data groups were analyzed with a one-way ANOVA, and post hoc analysis was performed using the Bonferroni procedure (IBM SPSS Statistics, v.28, IBM). The level of statistical significance was set at *p* < 0.05.

## Results

### Effects of changes in the focal depth on inspiratory-related signals

**Fig. 3:**
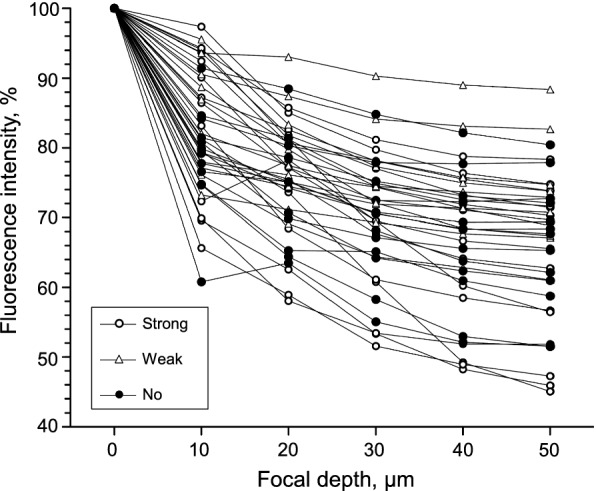
Changes in the baseline fluorescence intensity in each region at each depth. Each symbol indicates data obtained from the single ellipsoid region shown in Fig. 2A. The baseline fluorescence intensity was defined as a mean value of the averaged wave from − 1.5 to − 5.0 s triggered by the C4 inspiratory burst at each focal depth (see the Materials and Methods section). Ordinate: the percentage of the baseline fluorescence intensity at each focal depth to the baseline fluorescence intensity at a depth of 0 μm. Abscissa: the focal depth. Data from the strong (*n* = 15), *open circles*; weak (*n* = 10), *open triangles*; and no (*n* = 13), *filled circles* inspiratory-modulated groups are shown. In all regions, the baseline fluorescence intensity gradually decreased with increasing focal depth

In the first experiment, we examined the effects of the focal depth on inspiratory-related calcium signals in six preparations. There was a lot of debris composed of dead cell fragments on the cut surface of the preparation. When the focus was deepened about 5–15 μm from the surface, dark oval-shaped regions (i.e., live stained cells) were gradually visualized. We changed the focal plane back and forth and set the focus at which the number of stained cells with a clear outline was maximum. This focal plane was set at 0 μm in this study. At this depth, there was little debris, and the region identification was not difficult. The fluorescence images of the most successful recording example are shown in Fig. 2. In total, 38 regions (i.e., cells) were selected at 0 μm. The long diameter of these regions was 13.2 ± 3.2 μm; the short diameter was 10.6 ± 2.4 μm. When the focal depth was deepened at 10-μm steps, the fluorescence values in all regions gradually decreased (Figs. 2, 3). The regions at 0 μm became impossible to recognize at 20 μm, and other regions became visible. Most of the newly recognized regions at 20 μm did not overlap with those at 0 μm. The cell shape could not be distinguished when the focal depth was > 50 μm from 0 μm.

**Fig. 4:**
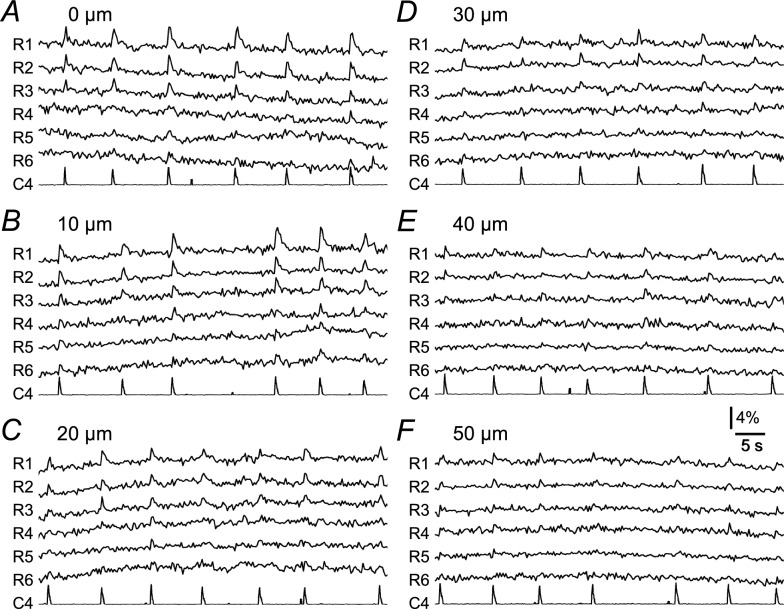
Representative raw records of fluorescence changes. **A**–**F** From top to bottom, the fluorescence signal in regions 1 to 6 (*R*1 to *R*6) shown in Fig. 2A, respectively, at each focal depth. The lowest trace (C4) represents the leaky integrated wave of electrical activity in C4VR. Vertical scale bar: 4% of the baseline fluorescence intensity for *R*1 to *R*6. The strong (*R*1 and *R*2), weak (*R*3 and *R*4), and no (*R*5 and *R*6) inspiratory modulation groups are shown

Figure 4 shows representative temporal changes in fluorescence intensity in the regions shown in Fig. 2A. We divided the regions into three groups based on the amplitude of the inspiratory-related fluorescence change at the focal depth of 0 μm: the ‘strong inspiratory modulation’ group included regions in which the amplitude of the inspiratory-related fluorescence change was > 2% and > 6SD of the baseline (*n* = 15); the ‘weak inspiratory modulation' group included regions in which the amplitude was ≤ 2% of the baseline but > 6SD of the baseline (*n* = 10); and the 'no inspiratory modulation’ group included the regions in which the amplitude was not greater than 2% or 6SD of the baseline (n = 13). The long diameter was 15.5 ± 2.6, 12.5 ± 2.7, 11.1 ± 2.6 μm for the strong, weak, and no inspiratory modulation groups, respectively. Similarly, the short diameter was 15.5 ± 2.6, 12.5 ± 2.7, 11.1 ± 2.6 μm. Both diameters in the strong inspiratory modulation group were significantly larger than those in the weak and no inspiratory modulation groups. Regions 1 and 2 (*R*1 and *R*2) in Fig. 4 were grouped in the strong inspiratory modulation group and showed clear inspiratory-related fluorescence changes at the focal depth of 0 μm (Fig. 4A). The amplitude of these inspiratory-related fluorescence changes in *R*1 and *R*2 gradually decreased as the focal depth was deeper (Fig. 4B–F). As shown in Fig. 4, the inspiratory-related fluorescence changes were difficult to see at the level of raw records in the regions of the weak inspiratory modulation group (*R*3 and *R*4) and those of the no inspiratory modulation group (*R*5 and *R*6).

**Fig. 5:**
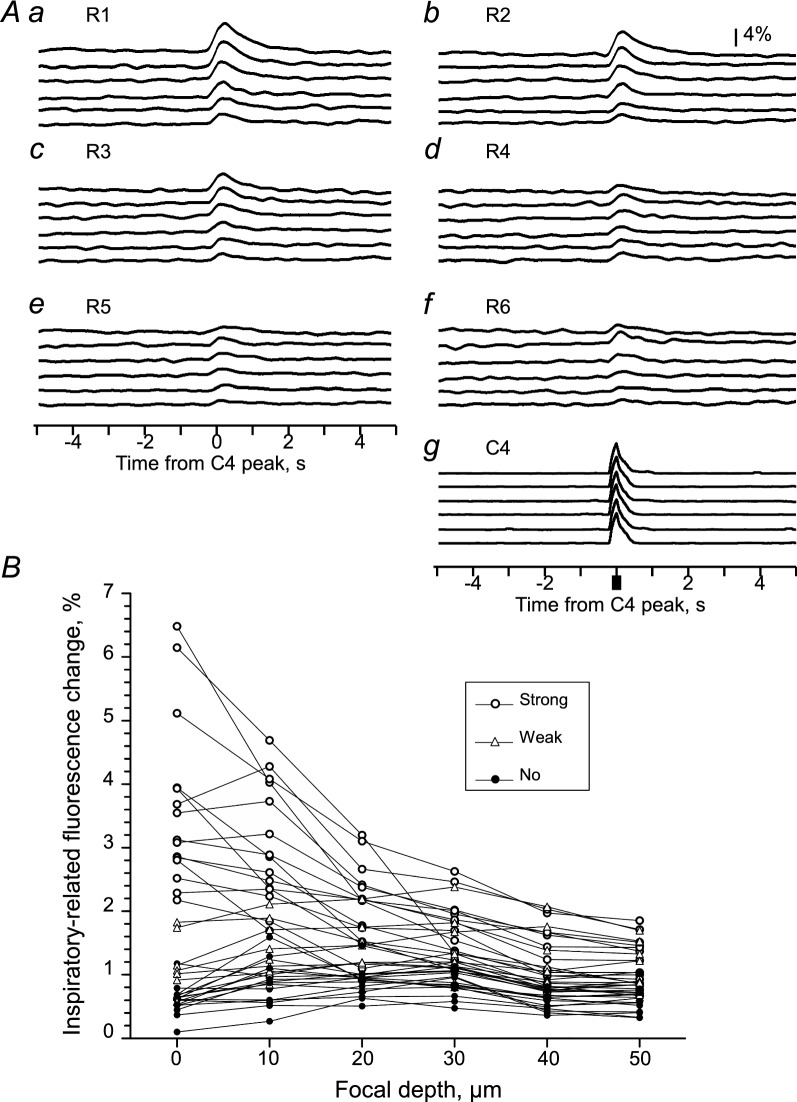
Representative example of the averaged fluorescence signal triggered by C4 inspiratory activity. **Aa**–**f** From top to bottom, each trace shows the average fluorescence signal obtained at each depth from the focus (0, 10, 20, 30, 40 and 50 μm), respectively, triggered by the C4 inspiratory activity in the strong (*R*1 and *R*2), weak (*R*3 and *R*4), and no (*R*5 and *R*6) inspiratory modulation groups. **Ag** From upper to lower, the averaged wave of leaky integrated C4 activity obtained at each depth from the focus (arbitrary unit). **B** Summary graph showing the amplitude of the inspiratory peak at each depth of focus. The data from the strong (*n* = 15), *open circles*; weak (*n* = 10), *open triangles*; and no (*n* = 13), *filled circles* inspiratory-modulated groups are shown. Note that the open circles gradually decrease with increasing depth, whereas the other symbols tend to be constant and at approx. 1% at any depth

Figure 5A provides the averaged data from Fig. 4. The amplitude of inspiratory-related fluorescence signals gradually decreased when the focal depth was deepened (Fig. 5Aa, b). This was applicable to all 15 regions of the strong inspiratory modulation group (blank circles in Fig. 5B). In all 10 regions of the weak inspiratory modulation group, the inspiratory-related fluorescence signal did not change as much with the deepening of the focal depth (Fig. 5Ac, Ad, blank triangles in Fig. 5B). In all 13 regions of the no inspiratory modulation group, although the amplitude of the inspiratory-related fluorescence signal was ≤ 6SD of the baseline at the focal depth of 0 μm, the amplitude was > 6SD of the baseline at one or more other focal depths (Fig. 5Ae, Af, filled circles in Fig. 5B). If the fluorescent calcium indicator stained only one cell, both the baseline fluorescence and the inspiratory-related fluorescence should decrease to the same degree with deepening of the focus, and the amplitude of inspiratory-related fluorescence signals should not change. However, since the fluorescent calcium indicator was injected into the thoracic spinal cord and many cells were stained, the component of the fluorescence from cells in the deep tissue increased in the baseline fluorescence with a deeper focus. As a result, these results suggest that the inspiratory-related fluorescence signal that decreases with the deepening of the focal plane in the strong inspiratory modulation group came from the focused cell. In contrast, other weak inspiratory-related fluorescence signals in the other two groups would come from other cells nearby in deeper focus. Similar results were obtained from all other five preparations examined.

### Effects of strychnine on the amplitude of the inspiratory-related calcium rise

In the second experiment, we examined the effects of strychnine on the inspiratory-related fluorescence signal in five preparations. Since strychnine at high concentrations blocks not only glycine receptors but also GABA_A_ receptors [12], we used 10 μm strychnine as in our previous study [2]. Strychnine did not eliminate the C4 inspiratory rhythm, but seizure-like activity was often observed [13, 14].

**Fig. 6:**
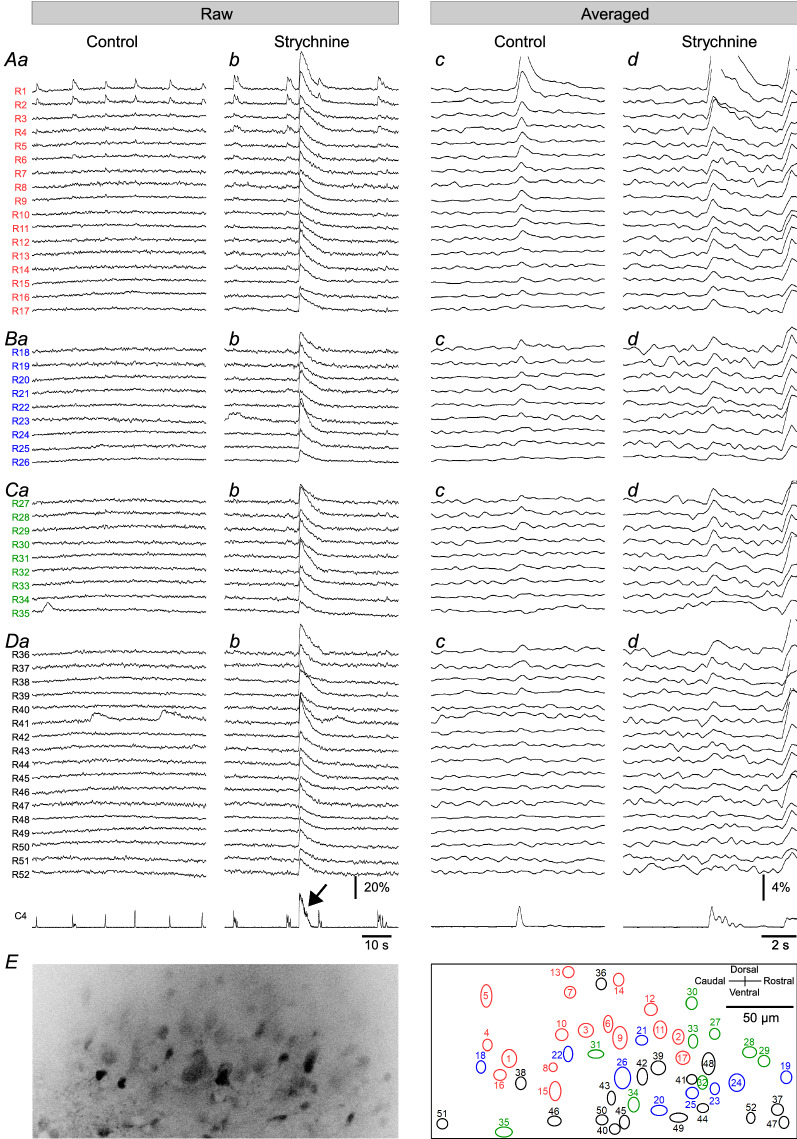
Effects of strychnine on the inspiratory-related fluorescence change. **A**–**D** From upper to lower, the data obtained from *R*1 to *R*52 and the leaky integrated C4 activity (C4). **a**, **b** Raw fluorescence changes from each region under control and strychnine, respectively. **c**, **d** Mean fluorescence change under control and strychnine, respectively. **A** Group of regions in which the amplitude of the mean inspiratory-related fluorescence change was > 6SD of the baseline under both control and strychnine. **B**,** C** Group of regions in which the amplitude was > 6SD of the baseline only under control or strychnine, respectively. **D** Group of regions in which the amplitude was < 6SD under both conditions. **E** *Left panel*, a fluorescence image from which the data of panels A–D were obtained. *Right panel*, the relative position of each region (*R*1–*R*52) to the left panel. Each region of each group in panels A–D is marked by an ellipse with *red*, *blue*, *green*, and *black lines*, and its region number is given inside or in the vicinity of the ellipse

Figure 6 shows the raw and averaged fluorescence signals in 52 regions under the control and strychnine conditions. The long diameter of these regions was 10.9 ± 2.5 μm; the short diameter was 8.4 ± 1.4 μm. We divided these regions into four groups depending on the effects of strychnine on the inspiratory-related fluorescence signal. In the first group, the amplitude of the average inspiratory-related fluorescence change was > 6SD of the baseline under both control and strychnine conditions (Fig. 6A, red ellipsis in Fig. 6E, *n* = 17). In the second and third groups, the amplitude of the average inspiratory-related fluorescence change was > 6SD only under the control condition or strychnine, respectively (2nd group: Fig. 6B, blue ellipses in Fig. 6E, *n* = 9; 3rd group: Fig. 6C, green ellipses in Fig. 6E, *n* = 9). In the fourth group, the change was < 6SD under both conditions (Fig. 6D, black ellipses in Fig. 6E, *n* = 17). The long diameter was 11.6 ± 2.9, 11.2 ± 2.6, 10.6 ± 1.5, 10.3 ± 2.3 μm for each group. Similarly, the short diameter was 9.0 ± 1.3, 8.8 ± 2.0, 7.9 ± 0.8, 7.9 ± 1.1 μm. Both diameters in each group were not significantly different between the groups.

In this visual field, three regions showed large inspiratory fluorescence changes (> 2% of the baseline) under control conditions (Fig. 6A, *R*1 = 6.57%, *R*2 = 4.63%, *R*3 = 2.12%). These three regions corresponded to the strong inspiratory modulation group described above, and the inspiratory-related fluorescence changes clearly increased under strychnine (*R*1 = 10.61%, *R*2 = 7.26, *R*3 = 3.07%). Similarly, in the other 14 regions of the first group, the application of strychnine significantly increased the inspiratory fluorescence changes (control, 1.28 ± 0.40%; strychnine, 2.04 ± 0.91%, *p* < 0.0001, *n* = 14).

We were especially interested in the third group that showed significant inspiratory changes under strychnine but not under the control condition (Fig. 6C), since this could imply the new recruitment of inspiratory cells under the blockade of glycine and GABA_A_ receptors. However, the increase in inspiratory fluorescence change was not large in all nine regions of the third group (mean increment = 0.82 ± 0.33%). In only *R*28, the inspiratory-related fluorescence changes exceeded 2% of the baseline under strychnine.

All regions showed large visible fluorescence changes during the seizure-like activity, regardless of the groups (Fig. 6 arrow, *R*1 = 33.84%, *R*2 = 28.32%, *R*3 = 19.42%, 1st group except *R*1–*R*3 = 17.23 ± 3.40%; 2nd group = 16.48 ± 6.88%; 3rd group = 19.29 ± 5.69%; 4th group = 17.70 ± 7.15%). Similar results were obtained from all other four preparations examined.

### Effects of the local bath application of strychnine to the spinal cord on the amplitude of the inspiratory-related rise in calcium

Since we applied strychnine to the entire preparation in the second experiment, glycine and GABA_A_ receptors were blocked not only in the spinal cord but also in the brainstem. Therefore, in the third experiment, we examined the effects of a local bath application to the spinal cord. We split the chamber at the first to third cervical segments and applied strychnine to the spinal cord.

**Fig. 7:**
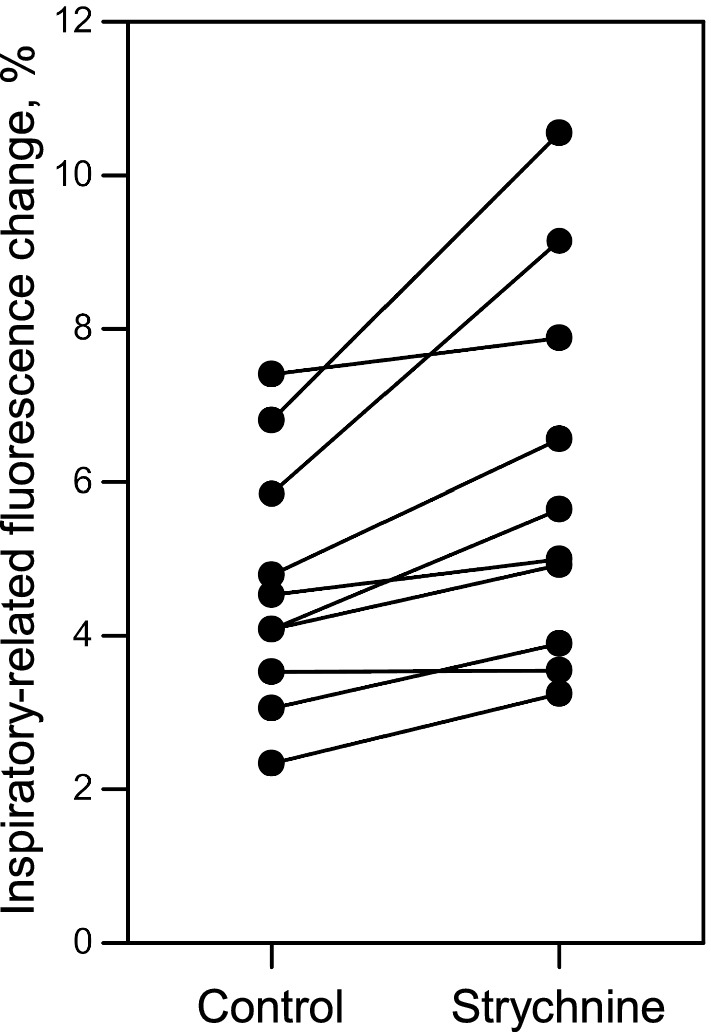
Effects of a local application of strychnine to the neonatal rat spinal cord on the inspiratory-related fluorescence increase. Each *filled circle* represents the peak value of the inspiratory-related fluorescence change under the control or strychnine condition obtained from one region. Data obtained from the same region are interconnected by a line. Although the degree of the increase depended on the region, no region showed a decrease in its inspiratory-related fluorescence change under strychnine. The fluorescence change increased significantly with strychnine (*p* < 0.01)

Since the results of the first experiment suggested that the inspiratory-related fluorescence signal with an amplitude > 2% of the baseline would come from the focused cell, we next analyzed only the regions that showed the inspiratory-related fluorescence signal with an amplitude > 2% of the baseline under control. In all 10 regions of the three preparations, strychnine significantly increased the inspiratory-related fluorescence change (Fig. 7, *p* < 0.01).

## Discussion

Using a fluorescent calcium indicator, we recorded the rise in inspiratory-related calcium in cells of the neonatal rat thoracic spinal cord. Since deepening the focus caused a decrease in inspiratory-related fluorescence in cells that showed inspiratory-related fluorescence changes with an amplitude > 2% of the baseline, we concluded that these inspiratory-related fluorescence signals come from the targeted cells. The blockade of glycine and GABA_A_ receptors by strychnine in the spinal cord increased the inspiratory-related fluorescence changes in these cells. This result suggests that a portion of these cells play a role in the control of the inspiratory motor output.

We next discuss (1) the technical difficulty in studying inspiratory cells in the spinal cord using calcium imaging with a confocal microscope; (2) the involvement of neurons and astrocytes in the inspiratory-related calcium rise, and (3) the intracellular calcium increase during seizure-like activity.

### Technical considerations

The spinal cord of the neonatal rat is rather transparent compared to the adult spinal cord, since myelination has not yet occurred. However, the cells at a deeper position (> 50 μm from the surface) were indiscernible in the present study. The confocal microscope thus does not appear to be suitable for studying the neuronal networks existing in deep nerve tissue, even in neonatal rats. In studies using two-photon or multiphoton microscopes [15, 16], cells in deep nerve tissue could be visualized more sharply even in the adult central nervous system. In the present experiments, although we tried to carefully remove the ventral funiculus using a fine insect pin, we were not able to record any respiratory activity from cells in the ventromedial region of the neonatal thoracic spinal cord in many preparations. We suspect that the spinal cord was pulled too much in those preparations, or the insect pin was inserted into the deep nerve tissue during the removal of the ventral funiculi. The use of a two- or multiphoton microscope thus seems preferable, since it would not be necessary to remove the ventral funiculus to record from individual cells in the medioventral region of the rat neonatal spinal cord.

Despite these technical difficulties, we were able to record the rise in inspiratory-related calcium from cells in the ventromedial surface of the thoracic spinal cord. This area corresponds to the area in which glutamatergic inspiratory spinal interneurons exist in neonatal rats [3].

Our results also revealed that deepening the focus dimmed the cell profile. Simultaneously, doing so decreased the inspiratory-related fluorescence changes in the cells that showed relatively large inspiratory-related fluorescence changes (i.e., > 2.0% of the baseline, the strong inspiratory modulation group). This result provides evidence that the fluorescence changes were from the focused cells. Although in many other regions there was a weak inspiratory-related fluorescence change (i.e., ≤ 2.0% of the baseline, the weak inspiratory modulation group), the amplitudes of the fluorescence changes were not as strongly affected by the focal depth. The range of inspiratory-related fluorescence changes at a depth of 50 μm was approx. 0.4–2.0% of the baseline (Fig. 5B). It would be difficult to determine to what extent the inspiratory-related fluorescence changes in the cells of the strong inspiratory modulation group come from neighboring cells.

Since the baseline fluorescence intensity decreased with focal depth (Fig. 3), the percentage of inspiratory-related fluorescence change from neighboring cells at a depth of 0 μm should be rather small. In the regions that showed an inspiratory-related fluorescence change < 2% of the baseline but > 6SD of the baseline under control conditions, strychnine significantly increased the inspiratory-related fluorescence change < 1% (control, 1.28 ± 0.40%, strychnine, 2.04 ± 0.91%, *p* < 0.0001, *n* = 14). Therefore, at least, the activity of two cells in Fig. 6 (*R*1, *R*2) and two other cells in Fig. 7 appeared to have increased under strychnine, since strychnine increased the inspiratory-related fluorescence changes to > 2% of the baseline in these cells at the depth of 0 μm. These results support our hypothesis that inspiratory interneurons play a role in enhancing inspiratory motor activity.

### Inspiratory-related calcium rise in neurons and astrocytes

The use of patch recording with solution containing the fluorescent dye Fura-2 in the electrode indicated that respiratory neurons in the brainstem slice of the neonatal mouse exhibited a calcium increase in phase with its action potential [17]. That study showed that the increase in intracellular calcium was approx. 50 nM with a single action potential and was approx. 200 nM during the respiratory rhythmic burst. In other calcium imaging studies of neurons, the action potential always caused an increase in intracellular calcium [6, 16, 18–20]. It is likely that the soma of a spinal interneuron also has voltage-sensitive calcium channels, and that firing causes an increase in intracellular calcium. The cells that showed a rise in inspiratory-related calcium in the present study are thus likely to have been comprised of mostly spinal interneurons.

We speculated that many subthreshold interneurons would be recruited by strychnine and that the inspiratory motor output would be enhanced as a result. However, we observed that strychnine did not seem to recruit many subthreshold cells, as the increase in the inspiratory-related fluorescence change was not so large in the group of cells that did not show the inspiratory-related fluorescence change under the control condition (Fig. 6C). The contribution of newly recruited inspiratory interneurons to the enhancement of inspiratory motor output under strychnine should thus not be expected to be high.

The cell-permeant Oregon Green labeled not only neurons but also astrocytes [6, 21]. Astrocytes in a mouse hippocampal slice preparation were demonstrated to show a spontaneous increase in intracellular calcium [21]. Another study indicated that not only neurons but also many putative astrocytes in the preBötC showed a respiration-related calcium rise [6]. In those studies, astrocytes were discriminated from neurons with the use of a red fluorescent dye, sulforhodamine 101 (S*R*101), as it has been reported that S*R*101 specifically labels the astrocyte [22]. However, another investigation indicated that the labeling of astrocytes by S*R*101 was not as strong and specific in brainstem slices compared to the hippocampus [23]. To the best of our knowledge, no studies have examined the use of S*R*101 for the specific staining of astrocytes in the spinal cord. More research is necessary to determine whether S*R*101 specifically labels astrocytes in the spinal cord and whether the astrocytes in the spinal cord undergo an inspiratory-related calcium rise.

### Intracellular calcium increase during seizure-like activity

A blockade of the glycine and GABA_A_ receptors in the brainstem–spinal cord preparation or specifically in the spinal cord has evoked spontaneous seizure-like motor activity [2, 24]. In the present study, all observed regions showed a large increase in the levels of fluorescence during the seizure-like activity evoked by strychnine. Neurons, as well as astrocytes in the cortex, showed increased intracellular calcium during a 4-aminopyridine (4-AP)-evoked seizure [25]. In that study, the onset of the intracellular calcium increase in the astrocytes was delayed by > 3 s from the onset of the intracellular calcium increase in the neurons [25]. We did not observe such a difference in the onset of the increase in fluorescence between regions in the present study (Fig. 6).

Although we did not discriminate between neurons and astrocytes, both types of cells should be labeled with Oregon Green. In addition, although the duration of the calcium increase during the reported 4-AP-induced seizure was longer in the neurons than in the astrocytes [25], no between-region difference in the time course of the fluorescence increase during seizure-like activity was evident in the present study (Fig. 6). Several possible explanations of these differences can be considered; one is the difference in the seizure induction method, and another is the difference between the cortex and the spinal cord. Examinations of the involvement of astrocytes in seizure-like activity are necessary for a better understanding of the pathophysiology of the seizures.

### Conclusions

Our present findings indicate that the glutamatergic inspiratory interneurons in the ventromedial portion of the neonatal rat rostral thoracic spinal cord could play a role in controlling inspiratory motor output. To determine whether the changes in fluorescence observed by confocal microscopy are from the focused cells, it is necessary to establish whether fluorescence changes decrease with the deepening of the depth from the focus.

## Data Availability

The data sets used and/or analyzed in this study are available from the corresponding author on reasonable request.

## Abbreviations

C4VR: Fourth cervical ventral root
GABA_A_: Gamma-aminobutyric acid type A
pFRG: Parafacial respiratory group
preBötC: Pre-Bötzinger complex
S*R*101: Sulforhodamine 101
VRC: Ventral respiratory column
